# Forward-viewing echoendoscope provides single sessional three biliary drainage routes in a patient with pancreatoduodenectomy

**DOI:** 10.1055/a-2589-0610

**Published:** 2025-04-29

**Authors:** Tesshin Ban, Yoshimasa Kubota, Shun Sasoh, Tomoaki Ando, Takashi Joh

**Affiliations:** 1 36884 Department of Gastroenterology and Hepatology, Gamagori City Hospital, Gamagori, Japan

In patients with pancreatoduodenectomy, echoendoscopic intubation to the hepaticojejunostomy site on the jejunal limb is necessary to visualize the perihilar area 1 . Therefore, intubation of a forward-viewing curvilinear echoendoscope (FV-CLS) may be considered when balloon enteroscopy-assisted cholangiopancreatography fails 1 2 3 . A dedicated partially covered self-expandable metal stent (PCSEMS) with an excellent anchoring system has been developed for use in endoscopic ultrasound-guided biliary drainage (EUS-BD) 4 5 . Herein, we present the initial FV-CLS intubation, which provided three single-session biliary drainage routes, in a patient with perihilar recurrence after pancreatoduodenectomy.

An 84-year-old woman who underwent pancreatoduodenectomy with modified Child reconstruction for stage IIB pancreatic cancer was referred to our hospital due to perihilar obstruction with a dilated biliary tree ( Video 1 ). Our biliary drainage strategy involved direct cannulation of the biliary anastomosis or EUS-BD via the anastomosis site or residual stomach, all of which were attempted in a single session using FV-CLS (TGF-UC260J; Olympus Medical Systems, Tokyo, Japan). The FV-CLS was advanced into the reconstructed alimentary tract. However, the left intrahepatic biliary branch was 3.1 mm in diameter on endosonography of the residual stomach ( Fig. 1 , Video 1 ), and the anastomosis was obscured owing to tumor involvement ( Fig. 2 , Video 1 ). Therefore, we selected EUS-BD via the anastomosis site as follows: a 19-gauge needle puncture with a 0.025-inch guidewire advancement to the left bile duct, a 4-mm balloon dilation, and a PCSEMS (Niti-S Spring Stopper, 8 mm/10 cm; Taewoong Medical, Gimpo, Korea) deployment ( Fig. 3 , Video 1 ). The patient’s clinical course was uneventful.

Forward-viewing echoendoscope provides single sessional three biliary drainage routes in a patient with pancreatoduodenectomy.Video 1

**Fig. 1:**
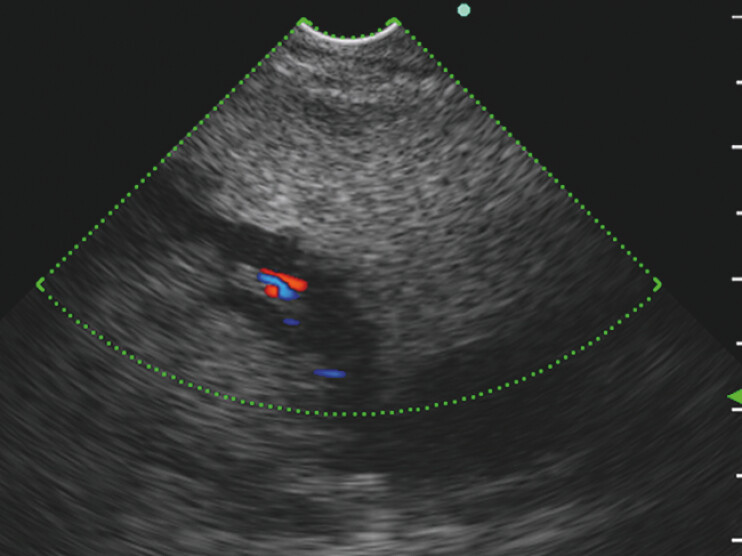
Left intrahepatic biliary branch depicted by forward-viewing curved linear echoendoscope (FV-CLS) from the residual stomach. Endoscopic ultrasonography-guided biliary drainage (EUS-BD) via the residual stomach was likely achieved; however, we hesitated to perform a puncture because the left intrahepatic biliary branch was narrow (3.1 mm in diameter).

**Fig. 2:**
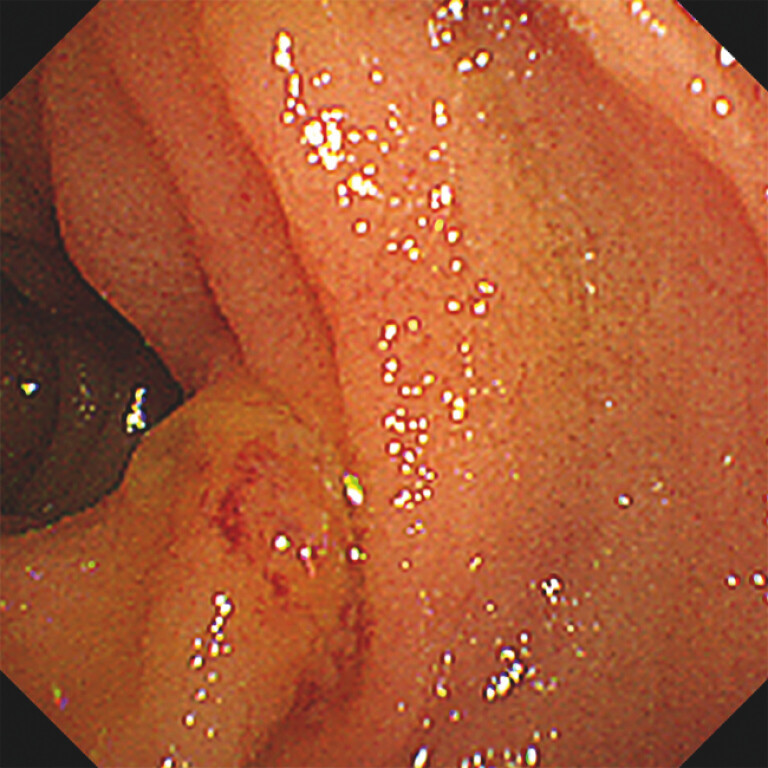
Biliary anastomosis area on the jejunal limb in the endoscopic view of FV-CLS. We sought a biliary anastomosis point for cannulation; however, this was challenging because of the deformity caused by tumor invasion.

**Fig. 3:**
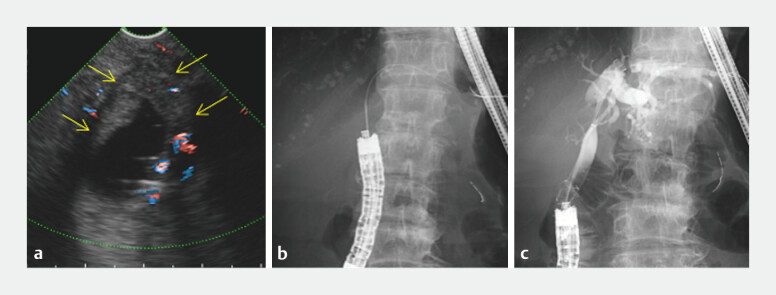
EUS-BD via the anastomosis site on the jejunal limb using FV-CLS. **a** FV-CLS revealed perihilar recurrence obstructing the dilated left hepatic duct after pancreatoduodenectomy. EUS-BD was feasible via the anastomosis site. Yellow solid arrows indicate the left hepatic duct involved in tumor recurrence. **b** A 19-gauge needle with a 0.025-inch guidewire was then advanced into the left hepatic duct. **c** A partially covered self-expandable metal stent with an antimigration system recanalized the obstruction.

Direct intubation with FV-CLS potentially provides three biliary access routes in a single session for patients with pancreatoduodenectomy and reconstruction, followed by biliary obstruction caused by perihilar recurrence: first, cannulation to the anastomosis if visible; second, EUS-BD via the afferent limb; and third, EUS-BD via the residual stomach.

Endoscopy_UCTN_Code_TTT_1AS_2AH

## Notes

Endoscopy E-Videos https://eref.thieme.de/e-videos
*E-Videos* is an open access online section of the journal *Endoscopy* , reporting on interesting cases and new techniques in gastroenterological endoscopy. All papers include a high-quality video and are published with a Creative Commons CC-BY license. Endoscopy E-Videos qualify for HINARI discounts and waivers and eligibility is automatically checked during the submission process. We grant 100% waivers to articles whose corresponding authors are based in Group A countries and 50% waivers to those who are based in Group B countries as classified by Research4Life (see: https://www.research4life.org/access/eligibility/ ). This section has its own submission website at https://mc.manuscriptcentral.com/e-videos .

## References

[1] Katanuma A Hayashi T Kin T Interventional endoscopic ultrasonography in patients with surgically altered anatomy: Techniques and literature review Dig Endosc 2020 32 263 274 31643105 10.1111/den.13567

[2] Hara K Okuno N Haba S Forward viewing liner echoendoscopy for therapeutic interventions Clin Endosc 2024 57 175 180 10.5946/ce.2023.271 38419166 PMC10984744

[3] Testoni PA Mariani A Aabakken L Papillary cannulation and sphincterotomy techniques at ERCP: European Society of Gastrointestinal Endoscopy (ESGE) Clinical Guideline Endoscopy 2016 48 657 683 10.1055/s-0042-108641 27299638

[4] Takasaki Y Isayama H Shin KS Measurement of the anchoring force of covered self-expandable and lumen-apposing metal stents for interventional endoscopic ultrasonography Dig Endosc 2023 35 96 102 10.1111/den.14406 35837746

[5] Ishii S Isayama H Sasahira N A pilot study of Spring Stopper Stents: Novel partially covered self-expandable metallic stents with anti-migration properties for EUS-guided hepaticogastrostomy Endosc Ultrasound 2023 12 266 272 37148139 10.4103/EUS-D-22-00104 PMC10237616

